# Supplementary material for: Causal Relationship between Aging and Anorexia Nervosa: A White-Matter-Microstructure-Mediated Mendelian Randomization Analysis
Source: Biomedicines. 2024 Aug 16;12(8):1874. doi: 10.3390/biomedicines12081874 (PMC11351342; doi:10.3390/biomedicines12081874)
Supplement: Supplementary file 1 [file biomedicines-12-01874-s001.zip › Supplementary Figure/Figure S4/Singe SNP plot of MR analysis for TL to ubm-b-2073.pdf]

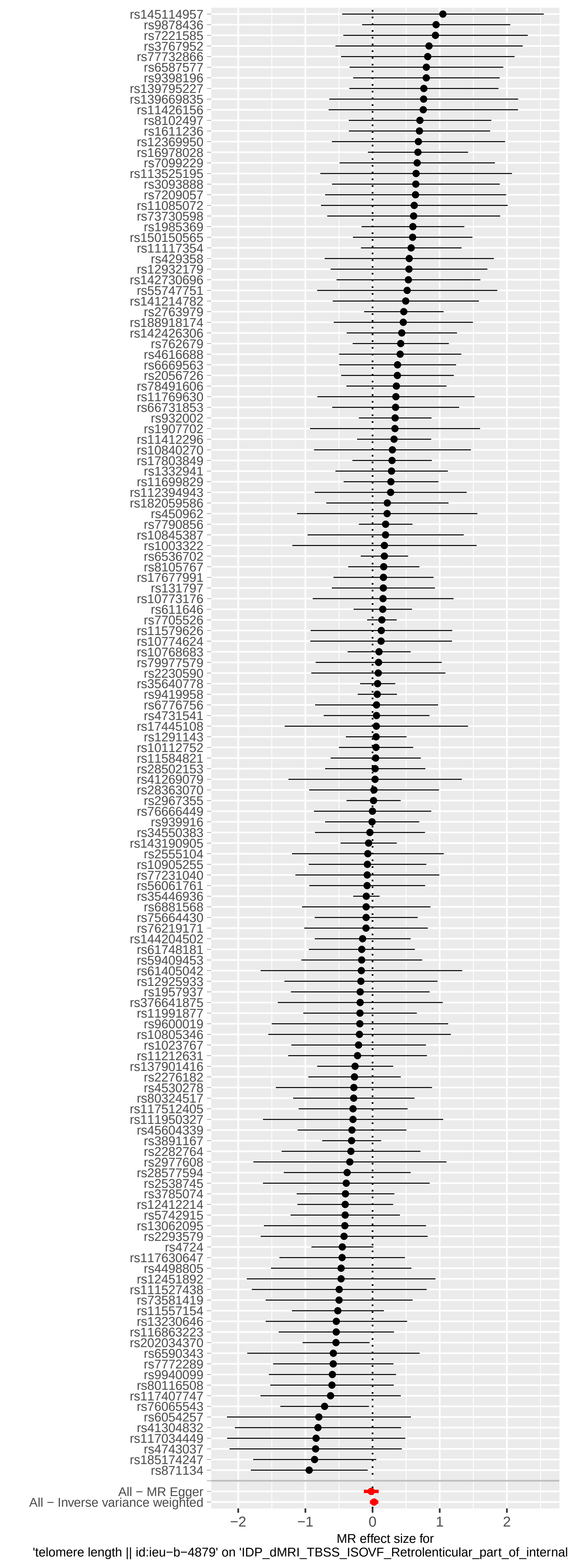
rs145114957
rs9878436
rs7221585
rs3767952
rs77732866
rs6587577
rs9398196
rs139795227
rs139669835
rs11426156
rs8102497
rs1611236
rs12369950
rs16978028
rs7099229
rs113525195
rs3093888
rs7209057
rs11085072
rs73730598
rs1985369
rs150150565
rs11117354
rs429358
rs12932179
rs142730696
rs55747751
rs141214782
rs2763979
rs188918174
rs142426306
rs762679
rs4616688
rs6669563
rs2056726
rs78491606
rs11769630
rs66731853
rs932002
rs1907702
rs11412296
rs10840270
rs17803849
rs1332941
rs11699829
rs112394943
rs182059586
rs450962
rs7790856
rs10845387
rs1003322
rs6536702
rs8105767
rs17677991
rs131797
rs10773176
rs611646
rs7705526
rs11579626
rs10774624
rs10768683
rs79977579
rs2230590
rs35640778
rs9419958
rs6776756
rs4731541
rs17445108
rs1291143
rs10112752
rs11584821
rs28502153
rs41269079
rs28363070
rs2967355
rs76666449
rs939916
rs34550383
rs143190905
rs2555104
rs10905255
rs77231040
rs56061761
rs35446936
rs6881568
rs75664430
rs76219171
rs144204502
rs61748181
rs59409453
rs61405042
rs12925933
rs1957937
rs376641875
rs11991877
rs9600019
rs10805346
rs1023767
rs11212631
rs137901416
rs2276182
rs4530278
rs80324517
rs117512405
rs111950327
rs45604339
rs3891167
rs2282764
rs2977608
rs28577594
rs2538745
rs3785074
rs12412214
rs5742915
rs13062095
rs2293579
rs4724
rs117630647
rs4498805
rs12451892
rs111527438
rs73581419
rs11557154
rs13230646
rs116863223
rs202034370
rs6590343
rs7772289
rs9940099
rs80116508
rs117407747
rs76065543
rs6054257
rs41304832
rs117034449
rs4743037
rs185174247
rs871134
All − MR Egger
All − Inverse variance weighted
−2
−1
0
1
2
MR effect size for
'telomere length || id:ieu−b−4879' on 'IDP_dMRI_TBSS_ISOVF_Retrolenticular_part_of_internal
